# Double plating versus nail–plate construct in AO 33C distal femur fractures: treatment choice affects knee alignment, clinical outcomes, and quality of life—a multicenter study

**DOI:** 10.1186/s10195-025-00834-1

**Published:** 2025-04-02

**Authors:** Domenico De Mauro, Amarildo Smakaj, Alessandro Casiraghi, Claudio Galante, Federico Bove, Mario Arduini, Giovanni Vicenti, Francesco Addevico, Abramo Fratus, Nicola Macellari, Matteo Caredda, Claudio Buono, Giulio Maccauro, Giuseppe Rovere, Francesco Liuzza

**Affiliations:** 1 Department of Orthopedics, Ageing and Rheumatological Sciences, Fondazione Policlinico Universitario Agostino Gemelli IRCCS, 00168 Rome, Italy; 2 Department of Orthopedics and Geriatrics Sciences, Università Cattolica del Sacro Cuore, Largo Francesco Vito, 8, 00168 Rome, Italy; 3 Department of Public Health, Orthopedic Unit, Federico II University, 80131 Naples, Italy; 4 Department of Biomedicine and Prevention, Orthopedic Unit, Tor Vergata University, 00133 Rome, Italy; 5 Orthopedics and Traumatology Unit, ASST Degli Spedali Civili, 25123 Brescia, Italy; 6 Orthopedics and Traumatology Unit, ASST Grande Ospedale Metropolitano Niguarda, 20162 Milan, Italy; 7 Orthopedics and Traumatology Unit, Ospedale Sant’Eugenio, 00144 Rome, Italy; 8 Department of Basic Medical Sciences, Neuroscience and Sense Organs, School of Medicine, Aldo Moro University, 70121 Bari, Italy; 9 Orthopedics and Traumatology Unit, Azienda Ospedaliero Universitaria Consorziale Policlinico “Giovanni XXIII”, 70124 Bari, Italy; 10 Department of Clinical Sciences and Translational Medicine, Orthopedics and Traumatology Unit, Tor Vergata University, 00133 Rome, Italy

**Keywords:** Distal femur fractures, Double plates, Plate and nail, Knee fractures, Complications

## Abstract

**Background:**

Distal femur fractures present a significant challenge for orthopedic surgeons, accounting for approximately 5% of all femoral fractures. Among the most commonly reported combined techniques in the literature are the double-plate technique and the retrograde nailing plus lateral plating, the nail–plate construct (NPC). The aim of our study is to compare surgical data, quality of life, and functional outcomes in 33-C fractures treated with either double-plate constructs or a retrograde nailing plus lateral plate.

**Materials and methods:**

A multicenter retrospective observational study was conducted in accordance with Strengthening the Reporting of Observational Studies in Epidemiology (STROBE) guidelines. Diagnoses were made on the basis of the AO classification, utilizing traditional radiological assessments. Patients were categorized into two groups on the basis of the surgical treatment they received: The NPC group comprised patients who underwent surgery with nail–plate construct, while the Plate group consisted of those who had surgery with double plating.

**Results:**

A total of 42 patients were included in the study. The NPC group comprised 26 patients with a mean age of 58.4 ± 18.8 years, while the Plate group consisted of 16 patients with a mean age of 61.3 ± 16.4 years. Significant differences were observed in knee extension recovery (*p* = 0.010) and lateral distal femur angle (LDFA) (*p* < 0.001). Linear regression showed a significant influence from treatment choice on all the Knee Injury and Osteoarthritis Outcome Score (KOOS) subscales, as well as in all domains of the European Quality of Life Five Dimensions Five-Level Version (EQ-5D-5L), except for the Daily Self-Care domain.

**Conclusions:**

Nail–plate constructs seems to lead to significantly better outcomes in AO type C distal femur fractures, compared with double plating, in terms of knee function and quality of life. Significant differences are shown also in anatomical outcomes, especially in extension gap, and LDFA.

*Level of evidence*: III

## Introduction

Distal femur fractures present a significant challenge for orthopedic surgeons, accounting for approximately 5% of all femoral fractures and 0.4% of all adult fractures, with an incidence of 8.7 per 100,000 people per year [1, 2]. These fractures typically show a bimodal distribution: they occur in young patients with good bone quality following high-energy trauma, and in elderly patients after ground-level falls, with a notable predominance in female individuals [3, 4].

Various classification systems exist for distal femoral fractures, with the AO/Orthopaedic Trauma Association (OTA) classification being the most widely used worldwide due to its comprehensiveness, reliability, and reproducibility [5, 6]. Our study employs this classification, focusing specifically on 33-C fractures, with articular surface involvement and different degrees of comminution (33-C1, 33-C2, and 33-C3). These complex fractures mostly require surgical intervention, and numerous treatment options are documented in the literature. However, consensus on the most effective treatment remains unclear. The use of devices such as lateral locking compression plates (LCP) and retrograde intramedullary nailing (RIMN) has largely prevailed over the older condylar screw and angled blade plate [7, 8]. Despite advancements, complication rates following these procedures remain high, with implant failure rates reaching up to 20% and non-union rates of up to 19% [9]. In particularly complex fractures, especially those with extensive metaphyseal comminution and osteoporotic bone, a single lateral LCP or RIMN may not provide adequate stable fixation and optimal healing environments, contributing to non-union or hardware failure [10, 11].

These challenges have led to the development of double fixation constructs. The rationale behind combined constructs is to offer more rigid fixation and enhanced stability at the fracture site, facilitating early mobilization and weight-bearing, which promote bone healing [12]. Among the most commonly reported combined techniques in the literature are the double-plate technique and the combined RIMN plus lateral LCP system, the nail–plate construct (NPC) [13, 14]. Studies have shown that adding a second medial buttress plate to the lateral LCP improves fixation stability and bone healing, with higher average loads to failure and construct survival rates compared with single lateral plating [15–17]. An alternative to the double-plate construct is the NPC, where a RIMN is supplemented by a lateral plate for added stability [18]. This construct spans the entire length of the femur, and by linking the nail and the plate distally, it facilitates easier force transfer at the bone–implant interface, resulting in better healing potential compared with lateral LCP or RIMN alone [19].

This technique combines the positive attributes of both implants, offering a balance of stability and micromotion that maximizes the probability of bone healing and early functional return [12]. Several studies have reported that nail–plate constructs in native and periprosthetic fractures of the distal femur achieve high union rates and low implant failure rates [18, 20, 21]. However, the literature still lacks comprehensive comparisons between these two combined techniques.

The aim of our study is (i) to compare surgical data, quality of life (QoL), and functional outcomes in 33-C fractures treated with either double-plate constructs or a RIMN plus lateral LCP system, and (ii) to analyze the impact of both techniques as factors affecting both functional and quality of life in this population.

## Materials and methods

### Study design

A multicenter retrospective observational study was conducted in accordance with Strengthening the Reporting of Observational Studies in Epidemiology (STROBE) guidelines [22], including patients from 2018 to 2022. The study followed the principles of the Declaration of Helsinki. The local institutional review board approved the study, with protocol number 0059449. All patients who participated in the study provided written informed consent prior to their inclusion, in accordance with ethical guidelines and regulatory standards.

### Inclusion and exclusion criteria

The inclusion criteria were (i) a diagnosis of distal femur fracture classified as 33-C according to the AO classification, (ii) an age of 18 years or older, and (iii) an indication for surgical treatment, either with double plating or NPC. Exclusion criteria included subjects with incomplete data, follow-up periods shorter than 9 months, periprosthetic fractures of the distal femur, open fractures, and pathological fractures.

### Data collection

Patient data were collected from registries of participating centers, including demographic information, medical and surgical histories, postoperative data, and mortality. Diagnoses were made on the basis of the AO classification, utilizing traditional radiological assessments with plain X-rays in anteroposterior and lateral views. When considered necessary by a senior surgeon, a second-level examination, such as a computed tomography (CT) scan, was performed. Surgical treatment decisions were made by a senior surgeon at each participating center, taking into account the fracture pattern, component stability, and the surgeons’ expertise. Patients were categorized into two groups on the basis of the surgical treatment they received: The NPC group included patients who underwent surgery with nail–plate construct, while the Plate group, consisted of those who had surgery with double plating.

### Surgical technique

Patients underwent surgery at participating centers, performed by senior surgeons experienced in complex articular fractures. Both types of surgery required the patient to be in a supine position, with an intraoperative image intensifier, and a tourniquet applied to the proximal part of the thigh. The primary objective for both groups was the anatomical reduction of the articular surface and then the addressing of the meta-diaphysis to restore the axis, angles, and rotation of the limb.

**Fig. 1:**
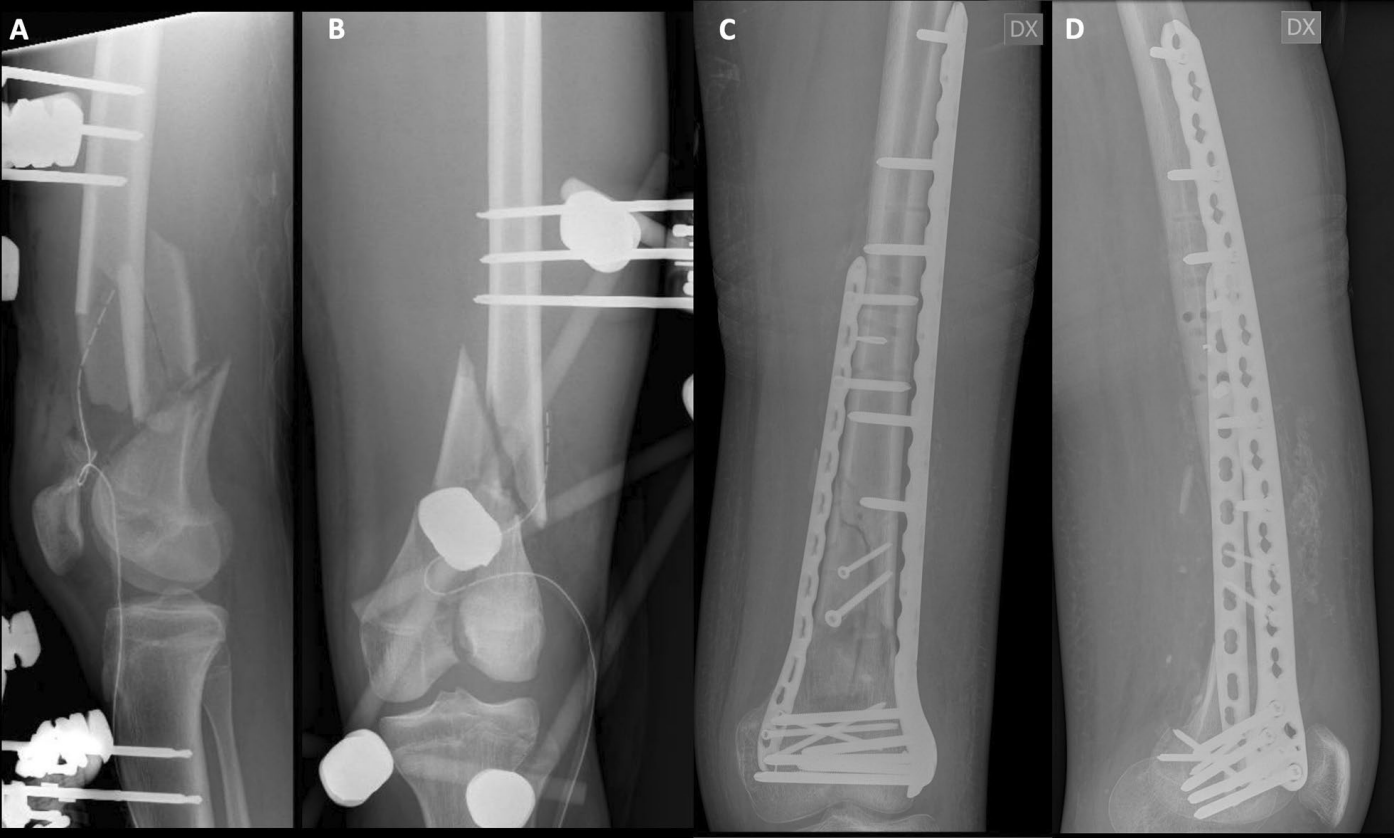
Preoperative radiographs in lateral view (**A**) and anteroposterior view (**B**), and postoperative radiographs in anteroposterior view (**C**) and lateral view (**D**) of a patient treated with double plating

**Fig. 2:**
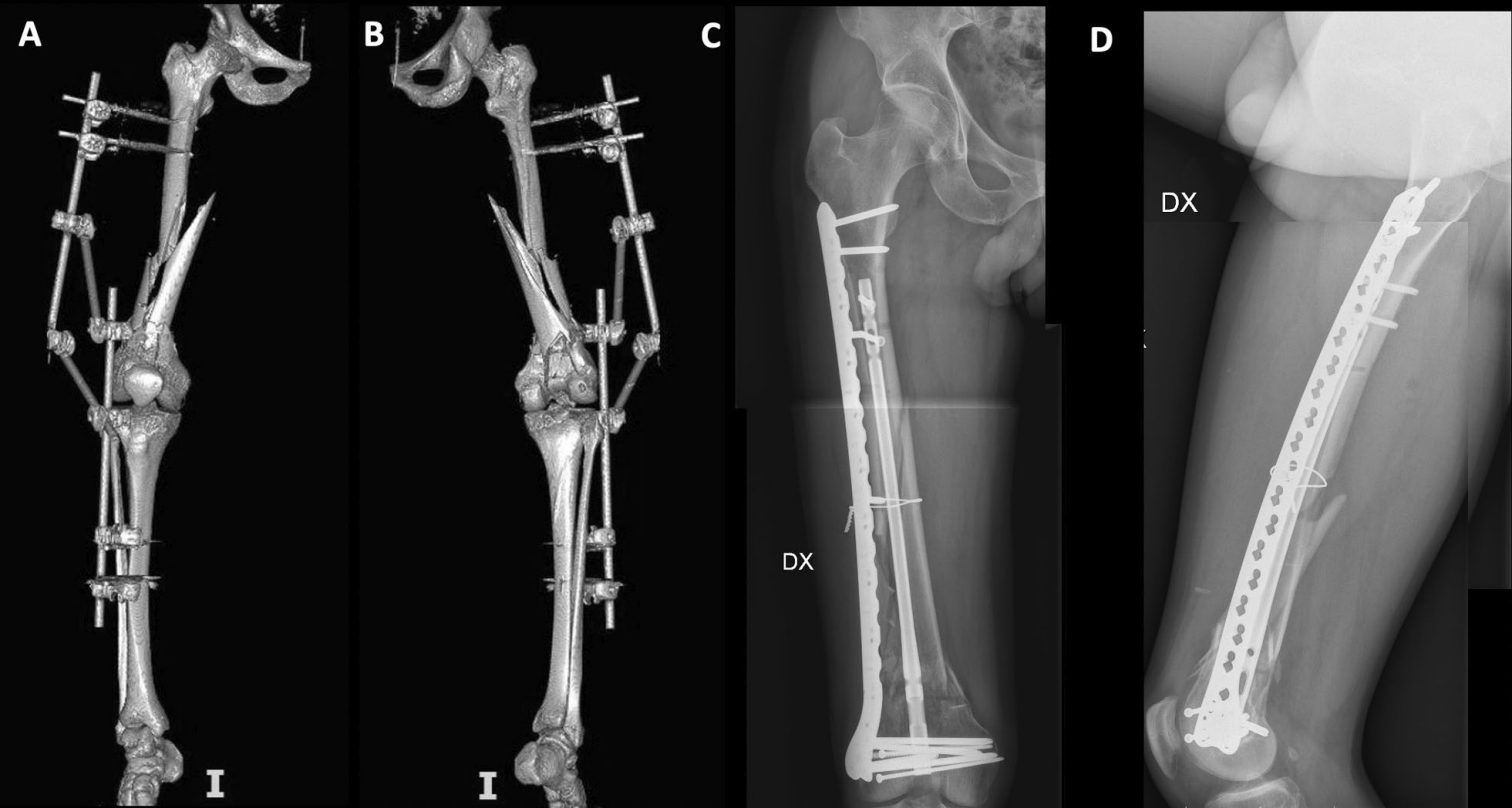
Preoperative three-dimensional (3D) CT reconstructions in anterior view (**A**) and posterior view (**B**), and postoperative radiographs in anteroposterior view (**C**) and lateral view (**D**) of a patient treated with a nail–plate construct (NPC)

In the Plate group, two different surgical approaches were used: lateral and medial. These approaches utilized soft-tissue-sparing techniques to minimize periosteal stripping and provide both mechanical and biological factors necessary for fracture healing (Fig. 1). In the NPC group, instead, two different techniques were employed. In 14 cases, a single approach was used, through a lateral parapatellar incision, as described by Krettek et al. [23]. In the remaining cases, instead, a double approach was chosen. The lateral incision remained the same, but a short incision centered on the patellar tendon was made to perform retrograde nailing of the femur. To achieve a better reduction prior to nailing, the lateral plate was preferentially implanted before the nailing procedure as per the surgeons’ preference (Fig. 2). Accurate hemostasis was achieved. The surgical wound was treated similarly in both groups.

### Postoperative assessment

Both groups followed the same postoperative protocol, which included early mobilization in bed and a progressive weight-bearing recovery during the follow-up period. Full weight-bearing was permitted at 6 weeks postsurgery. Follow-up appointments were scheduled at 2 and 4 weeks after surgery, and then at 3, 6, and 12 months. The final assessment for each patient was documented. A senior surgeon conducted the last evaluation for each patient, assessing clinical outcomes and administering a quality-of-life questionnaire.

**Fig. 3:**
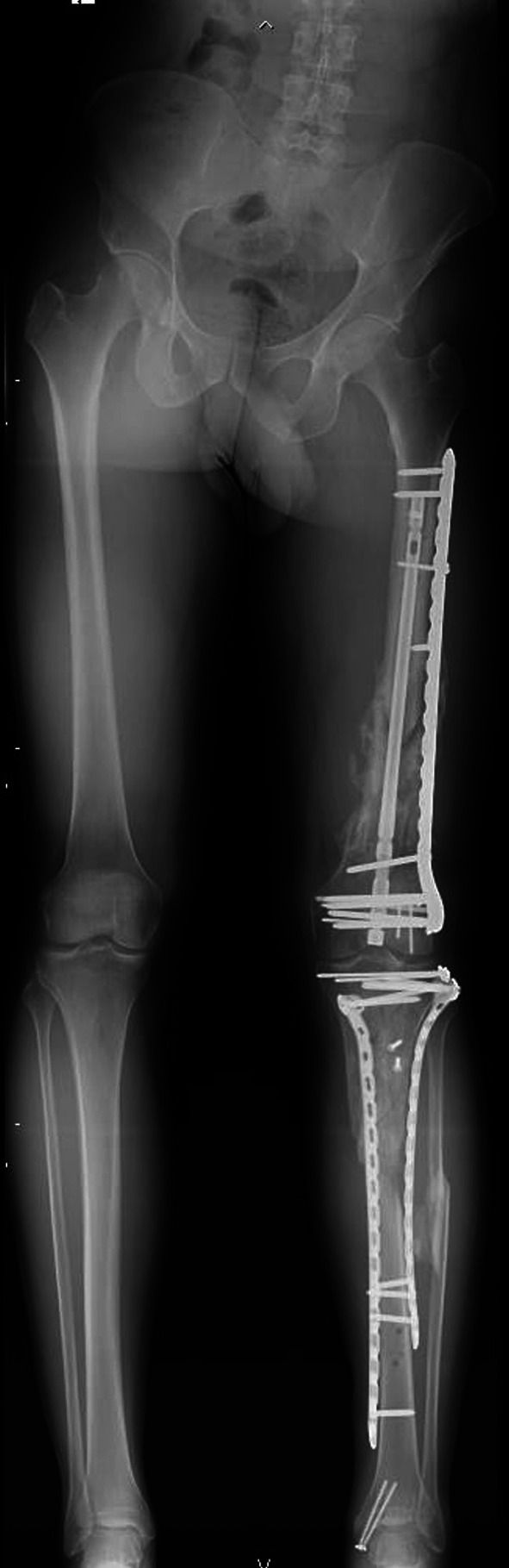
Example of a postoperative weight-bearing radiograph of the lower limbs from a patient in the NPC group, on which the lateral distal femoral angle (LDFA) and limb length discrepancy (LLD) were assessed by radiologists

A senior orthopedic surgeon conducted anatomical and radiological assessments taking into consideration clinical parameters such as rotational defects, range of motion of the affected knee (°), and leg length discrepancy (LLD) (mm). LLD and lateral distal femur angle (LDFA) were measured also through full-length standing X-Ray of the lower limbs by a radiology specialist (Fig. 3). The reference LDFA for arthritic patients was considered to be 88.1 ± 2.1°, while for healthy patients, it was 87.9 ± 2.1°, according to the findings from MacDessi et al. in their Coronal Plane Alignment of the Knee (CPAK) classification [24].

Clinical evaluation was conducted using the Knee Injury and Osteoarthritis Outcome Score (KOOS), a comprehensive self-reported questionnaire designed to assess short-term and long-term symptoms and function in patients with knee injuries and osteoarthritis. The KOOS consists of five subscales, with higher scores indicating better outcomes [25]. Quality of life was assessed using the European Quality of Life Five Dimensions Five-Level Version (EQ-5D-5L), a standardized measure of health status developed by the EuroQol Group. This tool evaluates five domains, each with five levels, where higher values correspond to poorer outcomes [26]. Complications, such as infections, non-unions, implant failures, were recorded, along with the reinterventions. The approach to managing complications varied depending on factors such as the surgeon’s experience, the type of complication, and the patient’s characteristics. The number of reinterventions exceeds the number of complications because some patients required multiple reinterventions for a single complication.

### Statistical analysis

The data are reported as the mean and standard deviation for parametric continuous variables, median and range for non-parametric continuous variables, and frequency distributions (%) for categorical variables. Continuous variables underwent comparison through either the Student *t*-test or the Mann–Whitney *U* test, depending on the distribution of the data. Categorical variables were represented as proportions and subjected to comparison utilizing either the Fisher exact test or the chi-squared test, accordingly. Linear regression analysis was employed to investigate the association between independent variables and dependent variables. Adjustments were made for potential confounding factors, to determine the independent effect of each predictor variable on the outcome of interest. The significance was established for a *p* < 0.05. SPSS 29.0 software program (SPSS, Inc., Chicago, IL, USA) was used.

## Results

A total of 42 patients were included in the study. The NPC group comprised 26 patients with a mean age of 58.4 ± 18.8 years, while the Plate group consisted of 16 patients with a mean age of 61.3 ± 16.4 years. Demographic data for both groups are detailed in Table 1. No significant differences were observed between the two groups regarding demographic data or preoperative and intraoperative variables, except for time to surgery (TTS) and the number of blood bags transfused. Additionally, there were no significant differences in hospitalization duration and follow-up length between the groups.

**Table 1:** Demographic data of included patients

	**NPC group**	**Control group**	***p*-Value**
*n*	26	16	
Age (years), mean ± SD	58.4 ± 18.8	61.3 ± 16.4	0.622
Female ratio, *n* (%)	11 (42.3%)	10 (63.5%)	0.204
BMI (kg/m^2^), mean ± SD	24.8 ± 3.8	27.1 ± 4.7	0.093
AO classification			
33–C1	4 (15.4%)	1 (6.3%)	0.375
33-C2	8 (30.8%)	5 (31.3%)	0.974
33-C3	14 (53.8%)	10 (62.5%)	0.582
Time-to-surgery (days), median (IQR)	11.5 (1–38)	5.5 (1–18)	0.008^*^^a^
Surgery time (minutes), median (IQR)	272.5 (108–645)	301.5 (112–657)	0.569^a^
Intraoperative blood loss (mL), median (IQR)	800 (350–2500)	700 (300–900)	0.342^a^
Blood transfused (number of bags), median (IQR)	4.5 (0–17)	1.0 (0–3)	0.001^*^^a^
Other procedures during the same surgery, *n* (%)	11 (42.3%)	8 (50.0%)	0.627
Hospitalization (days), median (IQR)	22.0 (7–78)	18.5 (4–58)	0.306^a^
Follow-up (days), mean ± SD	481.5 ± 185.6	507.6 ± 191.3	0.667

SD, standard deviation; IQR, interquartile range

^a^Mann–Whitney *U* test

**Fig. 4:**
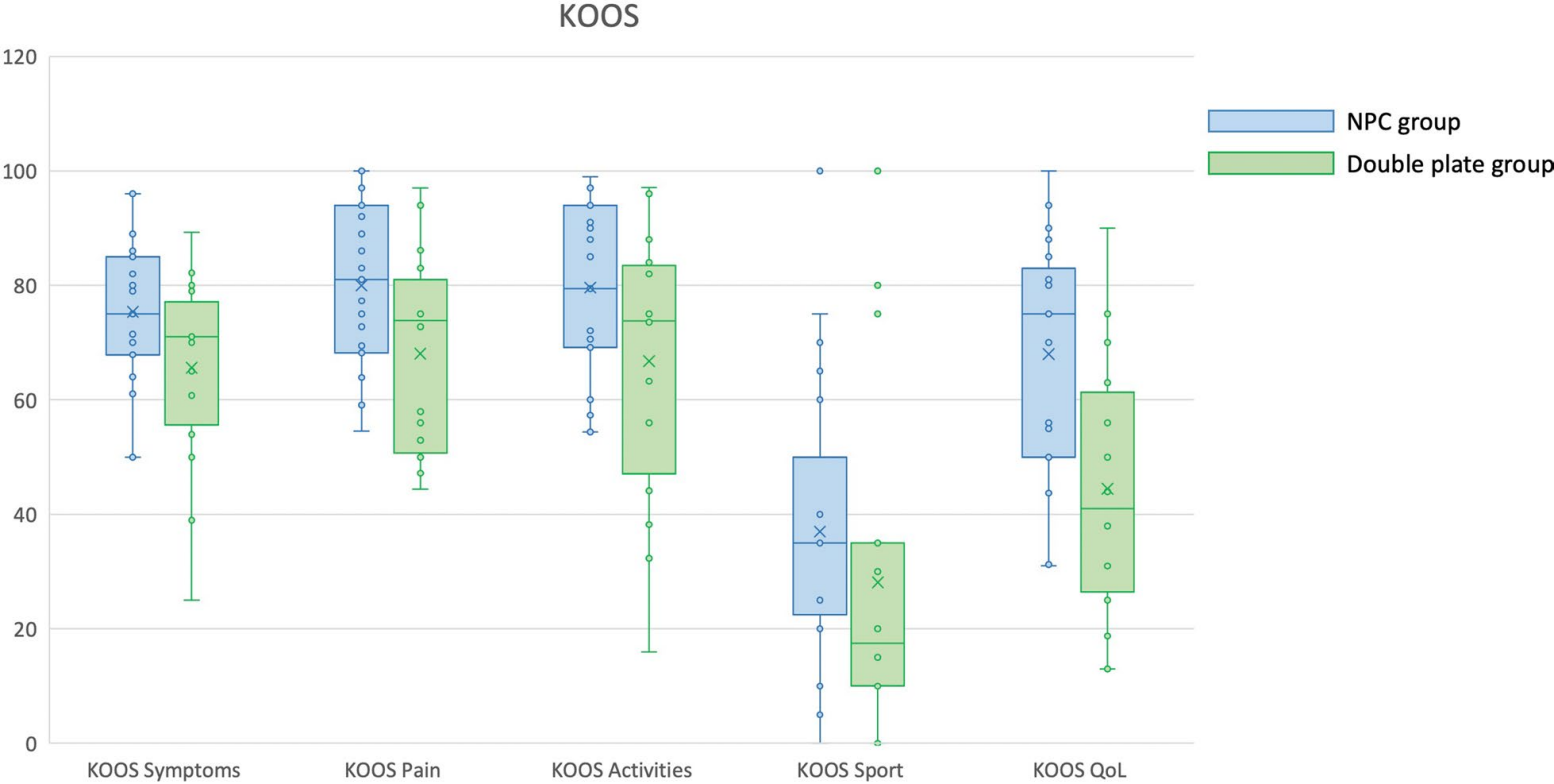
Differences between the two groups in terms of KOOS. NPC (light blue, on the left) shows significantly higher values than the double-plating group (light green, on the right) in all the items, defining better clinical outcomes in the NPC group. All the differences are statistically significant, with exception of for the value “Sports”

**Fig. 5:**
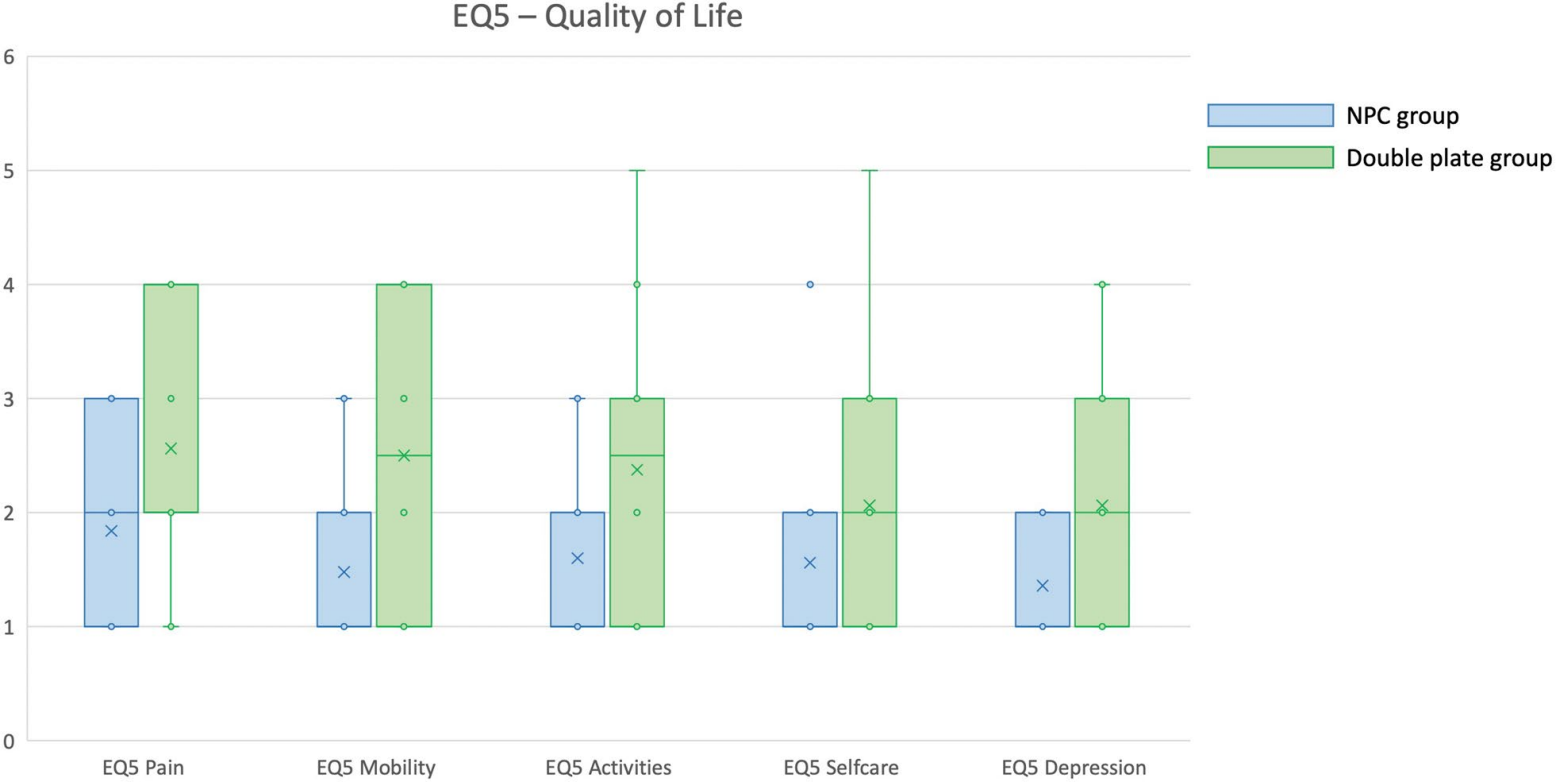
Differences between the two groups in terms of quality of life (EQ-5D score). NPC (light blue, on the left) shows significantly lower values than the double-plating group (light green, on the right) in all the items, defining higher quality of life in the NPC group. All the differences are statistically significant, with the exception of for the value “Self-care”

As a result of the surgical procedures, notable differences emerged between the two groups regarding anatomical outcomes, knee functional scores, and quality-of-life assessments, as shown in Table 2. Significant differences were observed in knee extension recovery (*p* = 0.010) and lateral distal femur angle (LDFA) (*p* < 0.001). Patients treated with double plating exhibited a greater extension gap and a more pronounced articular deformity compared with those in the NPC group. Mean KOOS values were generally higher in the case group. Significant differences were found in the Pain, Symptoms, Activities and Quality of Life subscales (*p* < 0.05; details can be found in Table 2; Fig. 4). Specifically, the European Quality of Life Five Dimensions (EQ-5D) score indicated that the NPC group performed better overall than the control group, particularly in the domains of mobility, pain, activities, and depression (*p* < 0.05; details can be found in Table 2; Fig. 5). There were statistically significant differences in complication rates between the two groups (*p* = 0.042).

**Table 2:** Comparison of postoperative outcomes between the two groups

	NPC group	Control group	*p*-Value
*n*	26	16	
ROM flexion (°), mean ± SD	103.0 ± 22.3	99.1 ± 15.9	0.523
ROM extension (°), mean ± SD	179.0 ± 2.0	176.8 ± 4.3	0.010^*^
LDFA (°), mean ± SD			
OA patients (target: 88.1 ± 2.1°)	89.6 ± 2.2	87.3 ± 2.4	0.040^*^
Non-OA patients (target: 87.9 ± 2.1°)	90.8 ± 2.4	86.5 ± 2.26	0.002^*^
Rotational defect, *n* (%)	2 (7.7%)	3 (18.8%)	0.283
LLD (mm), median (IQR)	0 (0–35)	10 (0–20)	0.059^a^
Knee Injury and Osteoarthritis Outcome Score (KOOS)			
KOOS symptoms, mean ± SD	75.4 ± 12.7	65.6 ± 16.6	0.035^*^
KOOS pain, mean ± SD	80.0 ± 13.7	68.0 ± 17.3	0.019^*^
KOOS activities, mean ± SD	79.6 ± 14.8	66.8 ± 23.5	0.037^*^
KOOS sports, median (IQR)	35.0 (0–100)	17.5 (0–100)	0.094^a^
KOOS quality of life, mean ± SD	60.0 ± 20.7	44.5 ± 22.8	0.002^*^
EQ-5D-5L quality of life			
Mobility, median (IQR)	1.0 (1–3)	2.5 (1–4)	0.008^*^^a^
Pain, median (IQR)	2.0 (1–3)	2.0 (1–4)	0.047^*^^a^
Activities, median (IQR)	1.0 (1–3)	2.5 (1–5)	0.032^*^^a^
Self-care, median (IQR)	1 (1–4)	2 (1–5)	0.149^a^
Depression, median (IQR)	1 (1–2)	2 (1–4)	0.026^*^^a^
Complications (overall)			
Complications (yes/no), *n* (%)	4 (15.4%)	7 (43.8%)	0.042^*^
Breakage, *n* (%)	0 (0.0%)	2 (12.5%)	0.065
Non-unions, *n* (%)	2 (7.7%)	1 (6.3%)	0.860
Infections, *n* (%)	2 (7.7%)	1 (6.3%)	0.860
Other complications^b^, *n* (%)	1 (3.8%)	3 (18.5%)	0.110
Reinterventions, *n* (%)	8 (30.8%)	5 (31.3%)	0.974

SD, standard deviation; OA, osteoarthritis; IQR, interquartile range; ROM, range of motion

^*^Statistically significant (*p* < 0.05)

^a^Mann–Whitney *U* test

^b^Other complications such as joint stiffness, malalignment, malunion, etc.

Upon analyzing LDFA based on the threshold values set by MacDessi et al. [24] for arthritic and healthy knees, 88.1 ± 2.1° and 87.9 ± 2.1°, respectively, results between the two groups were significant statistically: 89.6 ± 2.2° for the NPC group versus 87.3 ± 2.4° for the Plate group for arthritic knees, and 90.8 ± 2.4° versus 86.5 ± 2.26° for healthy knees.

To better understand the relationship between treatment choice and functional and quality of life outcomes, a linear regression analysis was conducted, adjusted for independent variables: age and sex. The results, presented in Table 3, indicate significant findings in both KOOS and EQ-5D-5L scores. NPC treatment demonstrated a strong positive influence on the Pain, Daily Activities, and Quality of Life subscales of the KOOS, as well as in all domains of the EQ-5D-5L, except for the Daily Self-Care domain. Conversely, logistic binomial regression did not lead to significant results regarding the influence of combined treatment on complication rates (*p* = 0.091).

**Table 3:** Univariate linear regression analysis, adjusted for age and sex

	**KOOS**					**EQ-5D**				
	Symptoms	Pain	Activities	Sport	QoL	Mobility	Pain	Activities	Self-care	Depression
*p*-Value	0.097	** < 0.001**	**0.020**	0.327	**0.001**	**0.006**	**0.003**	**0.031**	0.137	**0.009**
t*-Value*	−1.718	−3.695	−2.479	−0.999	−3.632	3.008	3.259	2.278	1.533	2.832
*95% CI*	−21.761	−23.516	−25.951	−26.470	−39.738	0.294	0.340	0.072	−0.177	0.199
	1.929	−6.724	−2.446	9.137	−11.046	1.558	1.496	1.389	1.226	1.246

	**Anatomical outcomes**									
	Extension gap	Flexion gap	Leg-length discrepancy	Knee alignment (LDFA)	Rotational defect					
*p*-Value	**0.009**	0.867	0.953	** < 0.001**	0.344					
t*-Value*	−2.800	−0.169	0.060	−4.190	2.710^a^					
*95% CI*	−6.014	−17.672	−6.912	−6.069	0.344					
	−0.927	14.980	7.329	−2.057	21.356					

Statistically significant *p*-values are highlighted in bold text

A significant impact of the surgical choice on knee postoperative anatomy, knee functional outcomes, and quality of life assessments, favoring the NPC treatment approach, was demonstrated. This finding underscores the superiority of the combined treatment in enhancing both the functional recovery and overall quality of life for patients. CI, confidence interval; OR, odds ratio

^a^This value represents OR, as rotational defect was a dichotomous variable, therefore a logistic regression was performed

## Discussion

The purpose of our study was to compare two surgical techniques, double plating and the nail–plate construct, in the treatment of type C distal femur fractures according to the AO classification. This comparison is particularly relevant, given the existing literature predominantly consists of case series and technical notes, with only three comparative studies, all of which are retrospective.

In the first comparative study by Passias et al. [27], 96 patients were included, but only 8 of these were treated with the NPC method. In contrast, our study includes over twice as many NPC cases (19 patients). Moreover, Passias et al. [27] did not clearly define the diagnosis, and only 38% of their cases involved the articular surface of the distal femur. Our study, in contrast, specifically addresses a homogeneous group of type 33-C fractures according to the AO classification, ensuring complete articular involvement in all cases.

The second comparative study by Shi et al. [28] compared NPC with single lateral plating rather than double plating as in our study. Additionally, the NPC group included 23 patients, but 4 of these were periprosthetic fractures, and only 55% were type C fractures according to the AO classification. This heterogeneity, which included both type A1 and type C3 fractures, complicates the interpretation of their findings.

The third comparative study by Garala et al. [29] also compared NPC with single lateral plating. Similar to the previous studies, the sample was heterogeneous, including periprosthetic fractures and both type A and type C fractures according to the AO classification. However, in the NPC group, the total number of non-periprosthetic fractures was 19, comparable to our study.

Our findings indicate that double plating results in greater extension deficits. A lower number of patients in the NPC group presented with loss of extension compared with the double-plating group, and additionally, the regression showed a direct correlation between treatment option and extension gap. This is a significant finding, as knee extension deficit is closely related to poorer outcomes in knee surgery, potentially leading to worse clinical outcomes [30, 31]. When assessing the LDFA, mean values differ significantly as per the MacDessi CPAK classification [24]. Our study highlighted a slightly higher value in the NPC group, indicating a minor variation toward varus alignment, especially in osteoarthritic patients. In contrast, patients treated with double plating showed a decrease in the LDFA (86°), indicating a more pronounced trend toward valgus alignment, which was significant in the analysis of differences between the two groups, and also in the linear regression, evidencing the role of the treatment choice as a risk factor in knee postoperative alignment. This finding highlights the significant impact of articular fracture treatments on knee alignment. It underscores the need for surgeons to consider the trend toward varus or valgus alignment when personalizing treatment options on the basis of the patient’s preoperative knee alignment. By tailoring the treatment approach to these alignment trends, surgeons can better manage surgical outcomes. Additionally, this understanding can aid in informing patients about potential postoperative changes in alignment and their implications, helping to set realistic expectations and improve patient care.

Moreover, patients treated with NPC demonstrated better outcomes in terms of knee function and quality of life. These differences were significant in both the comparative analysis between the two groups (*p* < 0.05) and in the linear regression analysis adjusted for age and sex. In terms of KOOS, the impact of NPC was significant, particularly concerning pain, daily activities, and overall quality of life, emphasizing the improved function of the joint. Moreover, in terms of the EQ-5D score, all subscales were significantly better in the NPC group. This included improvements in mobility, self-care, usual activities, pain/discomfort, and even the mental health dimensions related to the disease, such as depression and anxiety.

Our study has several limitations. Firstly, the sample size does not allow for strong, definitive statements about the data, and the retrospective design may lead to less robust conclusions. Moreover, the absence of prespecified criteria for treatment allocation may introduce selection bias, due to surgical decisions based on the senior surgeon’s expertise, fracture pattern, and component stability. However, given the lack of standardized guidelines in the literature, clinically based decision remains a crucial factor in managing these complex cases. Despite these limitations, our study offers several notable advantages.

Conducting a multicenter study has enabled us to collect more data, thereby enriching the sample size. Although our sample size is not very large, it is still one of the largest in the literature and certainly the largest when considering a homogeneous group of distal femur fractures, excluding periprosthetic fractures. Our study is among the very few comparative studies available, as most of the existing literature consists of case series. Within this limited group of comparative studies, ours stands out as the only one that focuses on a homogeneous diagnosis, type C fractures according to the AO Classification.

Additionally, our study is the only one that compares the nail–plate construct with double plating, rather than single plating. This comparison is valuable because double plating involves a more complex construct with two implants, making it a more appropriate counterpart to NPC than single plating. In conclusion, while the limitations of our study must be acknowledged, the strengths provide valuable insights and contribute to the existing knowledge.

## Conclusion

Nail–plate constructs seem to lead to significant better outcomes in AO type C distal femur fractures, compared with double plating, in terms of knee function and quality of life. Significant differences are shown also in anatomical outcomes, especially in extension deficit, and knee alignment. Further prospective studies with larger sample sizes and longer follow-up periods are necessary to confirm these findings and establish more definitive recommendations for clinical practice.

## Data Availability

The datasets used and/or analyzed during the current study are available from the corresponding author on reasonable request.

## Abbreviations

NPC: Nail–plate construct
LDFA: Lateral distal femur angle
LCP: Locking compression plates
RIMN: Retrograde intramedullary nailing
LLD: Leg length discrepancy
KOOS: Knee Injury and Osteoarthritis Outcome Score
TTS: Time to surgery
